# A concise history of skull base surgery: what is its contribution to neurosurgery?

**DOI:** 10.1007/s00701-025-06765-4

**Published:** 2026-01-17

**Authors:** M. Necmettin Pamir, Tiit Illimar Mathiesen, Zeynep Hüseyinoğlu, Baran Bozkurt, Koray Özduman

**Affiliations:** 1 School of Medicine, Department of Neurosurgery, Acıbadem University, Istanbul, Turkey; 2 Department of Clinical Medicine, University of Copenhagen, Copenhagen, Denmark; 3 Department of Neurosurgery, Rigshopitalet Copenhagen, Copenhagen, Denmark; 4 Department of Clinical Neuroscience, Karolinska Institutet, Stockholm, Sweden

**Keywords:** Skull-base, Microneurosurgery, Microneuroanatomy

## Abstract

**Background:**

Skull base neurosurgery (SBNS) emerged as a specialized branch of microneurosurgery as it addressed the challenges posed by intricate skull base anatomy. Initially developed through close collaboration with otolaryngologists, SBNS expanded in the 1990s and has undergone substantial advancements over the following decades. In this review, we analyze whether SBNS evolved as an organic development within neurosurgery or an external innovation, and we review key historical literature to support of either hypothesis.

**Methods:**

This is a synthetic, narrative historical review. An initial Pubmed review was performed with combination of keywords of “skull base neurosurgery”, “skull base surgery”, “neuroanatomy”, “microsurgical anatomy” and” neurosurgery complications”. Resulting database was restructured based on peer to peer, semi-structured interviews of two senior skull base neurosurgeons, with over 25 skull base neurosurgeons that were active 1970s–1990s. Emerging themes formed the framework for the analysis.

**Results:**

The evolution of SBNS was organic. It could be categorized into four distinct phases: Initially, Early attempts preceding the systematic application of SBNS techniques, subsequently the birth phase coincided with the widespread adoption of microneurosurgery and the establishment of dedicated societies and international meetings. During the popularization phase, advances in microneuroanatomy and novel approaches enhanced outcomes. Finally, the Maturation phase brought refined surgical approaches, the reevaluation of surgical indications, and the integration of stereotactic radiosurgery and endoscopic skull base surgery as well as international collaboration and teaching activities.

**Discussion:**

SBNS emerged within neurosurgery as a means to address challenging skull base pathologies and to enable surgical access through the skull-base. Its development was driven by collaboration with otolaryngology, alongside technological innovations such as the operating microscope, power drills, endoscopy, and stereotactic radiosurgery. These innovations facilitated the creation of novel surgical approaches, which were later refined through advances in neuroanatomical knowledge and improved understanding of pathology. Over time, SBNS were integrated into general neurosurgical practice and training curricula, allowing wide implementation and continued evolution in many directions.

##  Introduction

The skull base is an intricate anatomical region housing multiple cranial nerves and vascular structures within a framework of bone, cartilage, and dural folds. Unlike calvarial pathologies, skull base lesions are concealed beneath the brain and confined within complex compartments, often entangled with vital structures, presenting unique surgical challenges. The skull base accommodates a diverse spectrum of pathologies, including benign conditions (e.g., fibrous dysplasia), congenital anomalies (e.g., encephaloceles), trauma-related pathologies (e.g., skull base fractures), vascular disorders (e.g., aneurysms, cavernomas, arteriovenous malformations), and tumors (e.g., meningiomas, schwannomas, pituitary adenomas, chordomas, metastases, and malignancies extending from craniofacial carcinomas or hematological malignancies).

SBNS primarily targets benign, slow-growing tumors or vascular pathologies affecting cerebral circulation. Initial attempts were usually small personal series to tackle specific problems in neurosurgery. Close collaboration with otolaryngology, which already had a strong momentum treating skull base pathologies, led to general popularization of the SBNS concept within the field of neurosurgery. Within a few decades microneuroanatomical knowledge grew exponentially, leading to development of numerous novel approaches, most of which were elaborate, complicated and risky. Over time, these approaches were refined, standardized, and collectively recognized as "skull base approaches." For vascular lesions, skull base approaches provide optimal angles of access, reducing or eliminating the need for brain retraction. In the case of “skull base tumors”, these approaches enhance visualization, minimize surgical distance, and improve resection efficiency and safety, often allowing for prolonged disease-free and symptom-free intervals for patients.

Today, SBNS is not considered an esoteric surgical exercise, but on the contrary, an integral part of neurosurgery, a part of the standard curriculum to address a group of intracranial pathologies. Nevertheless, despite publication of several hundreds of books and over 13,000 Pubmed referenced articles, no comprehensive framework exists in the literature for charting the emergence of SBNS and its integration into the broader neurosurgical discipline. This review was prompted by the hypothesis that SBNS arose organically from within neurosurgery rather than as an external innovation. Our analysis demonstrates that SBNS matured through a series of overlapping yet distinguishable temporal phases before ultimately becoming an essential component of the neurosurgical curriculum.

## Methods

This work is a synthetic, narrative historical review aimed at tracing the evolution of skull base surgery, with an emphasis on major milestones, pioneering figures, and technological advances. A comprehensive literature review was conducted using electronic databases, primarily PubMed, covering publications from 1913 to 2025. Keywords were combinations of “skull base neurosurgery (13,975 results)”, “skull base surgery (21,497 results)”, “neuroanatomy (13,945 results)”, “microsurgical anatomy (8,728 results)” and “neurosurgery complications (141,860 results)”. Studies were selected based on their documentation of significant innovations, landmark procedures, or turning points in the field’s progression. When no supporting Pubmed listed articles were found on such mutual historical milestones, additional conference proceedings and books were added to the database.

The resulting database was filtered based on semi-structured, peer to peer interviews of two senior skull base neurosurgeons of the current manuscript (M.N.P. and T.I.M.) with 25 skull base neurosurgeons who had direct experience or historical knowledge relevant to the evolution of skull base surgery in the 1970–1990 period. Names of these 25 informant neurosurgeons are listed in the acknowledgements section. Selection criteria included recognized clinical or academic contributions to the field, active practice during key developmental periods, or authorship of landmark publications. These interviews were all conducted peer to peer either during fellowship trainings of the authors or during invited lectures of the corresponding skull base neurosurgeons at the institutions of the senior authors (Examples of these meetings include “The meningioma meeting at Marmara University-Istanbul-Turkey in 1990” for Laligham Sekhar; “The 10th Anniversary Scientific Meeting of Neurosurgery Department, Marmara University, Faculty of Medicine, Çırağan Palace, Istanbul, 1996” for Bernard George; “The Skull Base Surgery Meeting, Çırağan Palace, Istanbul, 1997 for Akira Hakuba, Ossama Al-Mefty, Chandranath Sen). The interviews were semi-structured and focused on milestones in technique development, conceptual shifts, and educational influences. No ethical review or consent was obtained during these collegial interviews. Interviews were not audio-recorded. Notes were taken during the interviews, and the responses were retrospectively and thematically analyzed to identify recurring concepts and historical trends.

A hermeneutic narrative was formed where independent observations and overarching explanatory models agreed and supported each other. A historical framework was formed based on mutual agreement between the two senior authors (MNP, TIM), which was supported by selected literature from the initial database. Finally, we evaluated whether the narrative supported a hypothesis of an intrinsic organic evolution within neurosurgery or a paradigm shift in terms of a scientific revolution. As this study is a literature-based historical review, ethical approval and patient consent were not applicable. The analysis was based on a combination of interview data, published literature, and archival sources. Potential limitations include the subjectivity of the interviews, and the non-systematic nature of qualitative data collection.

Our review is narrative rather than systematic, which introduces inherent selection bias and may overemphasize studies aligning with our expertise and perspective. Additionally, because our search and citations were limited primarily to English-language sources, important non-English contributions may have been underrepresented, potentially affecting completeness and generalizability.

## Results

### The time course of SBNS

When analyzed throughout its timeline, four distinct but overlapping phases with distinct characteristics can be distinguished: Early attempts, the birth, the popularization, and the maturation. The first phase, “early attempts” was characterized by individual and sporadic efforts of master neurosurgeons to perform surgery of the skull base. The second phase, saw systematic and organized efforts to use and develop the skull base approaches. Approaches that were sporadically used in the first phase, were systematically utilized, improved and novel approaches were devised. Early results of these systematic efforts were discussed in international meetings. Societies, federations, publications and frequent meetings disseminated the effort internationally. As this second phase brought general recognition of the term “skull base neurosurgery” within the international community, we chose to name it the “birth of SBNS”. As in most medical fields, the next phase saw frequent and liberal utilization of skull base approaches for various pathologies and therefore an accelerated development of novel approaches parallel to the similarly fast advances in microsurgical neuroanatomy, improved understanding of pathology. Therefore, we chose to call this phase the “popularization phase”. The latest and current phase is characterized by the refinement of approaches, the reevaluation of surgical indications, and the integration of stereotactic radiosurgery and endoscopic skull base surgery. In this latest phase international academic collaboration and teaching activities became so common that SBNS teaching is now easy to reach. With improved understanding, standardized clinical decision making, standardized surgical approaches and widespread teaching, various SBNS approaches became a part of the core curriculum in neurosurgery. Therefore, we chose to name it the “maturation phase” of neurosurgery. In the following sections we will give a synopsis of key developments in these four distinct phases.

### Phase one: early attempts

The earliest documented attempt at resecting a skull base tumor was performed by Francesco Durante in 1884, who successfully removed an olfactory groove meningioma through *transbasal approach*, achieving long-term survival and establishing one of the first milestones in skull base surgery [35]. His operation demonstrated the feasibility of accessing deep midline skull base regions, setting the conceptual groundwork for later *trans-sphenoidal procedures*. The earliest surgical attempts at addressing skull base pathologies primarily targeted the parasellar region. The pioneering efforts can be attributed to Herrmann Schloffer, who performed the first transsphenoidal surgery for pituitary adenoma resection via a lateral rhinotomy in 1907 [126]. This was followed by Oskar Hirsch in 1910, who introduced the *transseptal, submucosal transsphenoidal approach* [84]. In the same year, Albert E. Halstead devised the sublabial, gingival incision to access the sphenoid sinus [84]. Alternative routes were also explored, including Ottokar Chiari’s “*trans-ethmoidal”* and Hermann Preysing’s “*trans-palatal approaches”* [84]. Over subsequent decades, technological advancements such as improved illumination by Norman Dott, fluoroscopy by Gerard Guiot, and the introduction of the operating microscope significantly enhanced transsphenoidal surgery [84].

Following the initial successes with pituitary adenoma resections, skull base procedures expanded to craniofacial surgeries. Walter Dandy performed an orbital tumor resection via the anterior fossa, extending into the ethmoid bone [27]. This was the first documented attempt of craniofacial surgery. Dandy’s attempt was followed by Bronson S. Ray and John M. McLean’s combined transcranial and transorbital resection of a retinoblastoma in 1943 [119]. The evolution of craniofacial resections continued with Klopp, Smith & Williams attempt at resection of a frontal sinus cancer in 1954 [135] and Jan Malecki’s craniofacial approach for ethmoid carcinoma in 1959 [89]. The same period marked Patrick J. Derome and Gerard Guiot’s systematic development four distinct approaches to the spheno-clival region mostly to address craniofacial abnormalities [28].

### Phase two: the birth of systematic SBNS

The development of SBNS was deeply intertwined with two inventions: The advent of the operating microscope and the high-speed drill.

The first use of an operating microscope in surgery is credited to Swedish otologist Carl Nylen in 1921 [31] with its neurosurgical application introduced by Theodore Kurze in 1957 [143] James Gardner, Lawrence Pool, and Ernest Sachs further advanced its implementation [9, 152].A major breakthrough came in 1969 when M. Gazi Yaşargil introduced the counterbalanced, floating stand for the operating microscope and facilitated its systematic use in all neurosurgical procedures [146]. The widespread adoption of microneurosurgery led to significantly reduced surgical mortality and morbidity, as well as improved tumor resection rates. The use of the microscope also slowly opened the way for skull base approaches.

Bone removal to gain access to skull base structures is fundamental to SBNS. High-speed drills were first introduced by otolaryngologists. Initial drills were, however, mechanical lower speed dental instruments. The first use of a drill to remove external auditory canal hyperostosis was done by Arthur Mathewson in 1876 [93]. John Iseman [107] introduced a handheld air turbine in 1941 which was followed independently by George F. Green in 1945 and John Victor Borden in 1946 and Ivar Norlen in 1948 [62] [137].The first vane-type pneumatic drill, which later became the Midas-rex, was introduced in 1967 [109, 139].The extradural drilling of the sphenoid wing during the standard pterional craniotomy, as described by M.Gazi Yaşargil in 1969, is considered by many as a milestone marking the start of SBNS in its second phase [146]. During the 1990 s, the underlying rationale for the extensive bone removals that characterized skull base approaches was to minimize brain retraction when accessing deep regions of the skull base. Ossama Al-Mefty quantified the increased exposure without the need for brain retraction and coined the motto “remove the bone, leave the brain alone” [136] This motto was embraced by all skull base neurosurgeons and it was perhaps the single most important concept that guideded the develoment of SBNS.

Despite the adoption of these two inventions, extradural SBNS approaches were seldom attempted in neurosurgery before the 1990’s. Otolaryngologists played a pivotal role in popularizing such techniques. Prominent figures such as William House and Ugo Fisch developed alternative surgical routes for vestibular schwannomas, glomus tumors, and chordomas [147]. William House’s “*translabyrinthine approach”* provided a paradigm shift for popularization and widespread use of skull base surgery techniques [60]. Over the years, close interdisciplinary collaboration of neurosurgeons with otolaryngologists catalyzed the adoption of skull base techniques in neurosurgery [38]. A notable example is the work of neurosurgeon Madjid Samii and otolaryngologist Wolfgang Draf, who collaborated on techniques to preserve the facial nerve during “*retrosigmoid transmeatal”* vestibular schwannoma surgery [33]. With growing interest in the field, SBNS societies and congresses were established. The establishment of these skull base societies led to international recognition of the field. The first international skull base congress was organized by Madjid Samii in Hannover in 1992 [123]. With collaborations from national skull base societies the World Federation of Skull Base Surgery was founded and Madjid Samii became the first president.

### Phase three: popularization of SBNS

By the early 2000 s, SBNS had gained widespread recognition and acceptance. This third period started in the 1990-s and saw substantial progress in four key areas: First of all, the development of microneurosurgery significantly improved surgical outcome with increased extent of resections and much lower complication rates. Then, during this popularization phase, due to collective effort of international surgeons, the pathophysiology and the natural history of special entities (such as cavernous sinus meningiomas, trigeminal schwannomas etc.) were better recognized. Again, with the international collective effort, skull-base approaches were refined to make them faster, more effective and safer. Finally, the fourth characteristic was a shift from stereotypical/standard approaches to patient centered strategies and patient-customized surgical approaches.

#### SBNS and microneurosurgical anatomy kindled each other

**Fig. 1:**
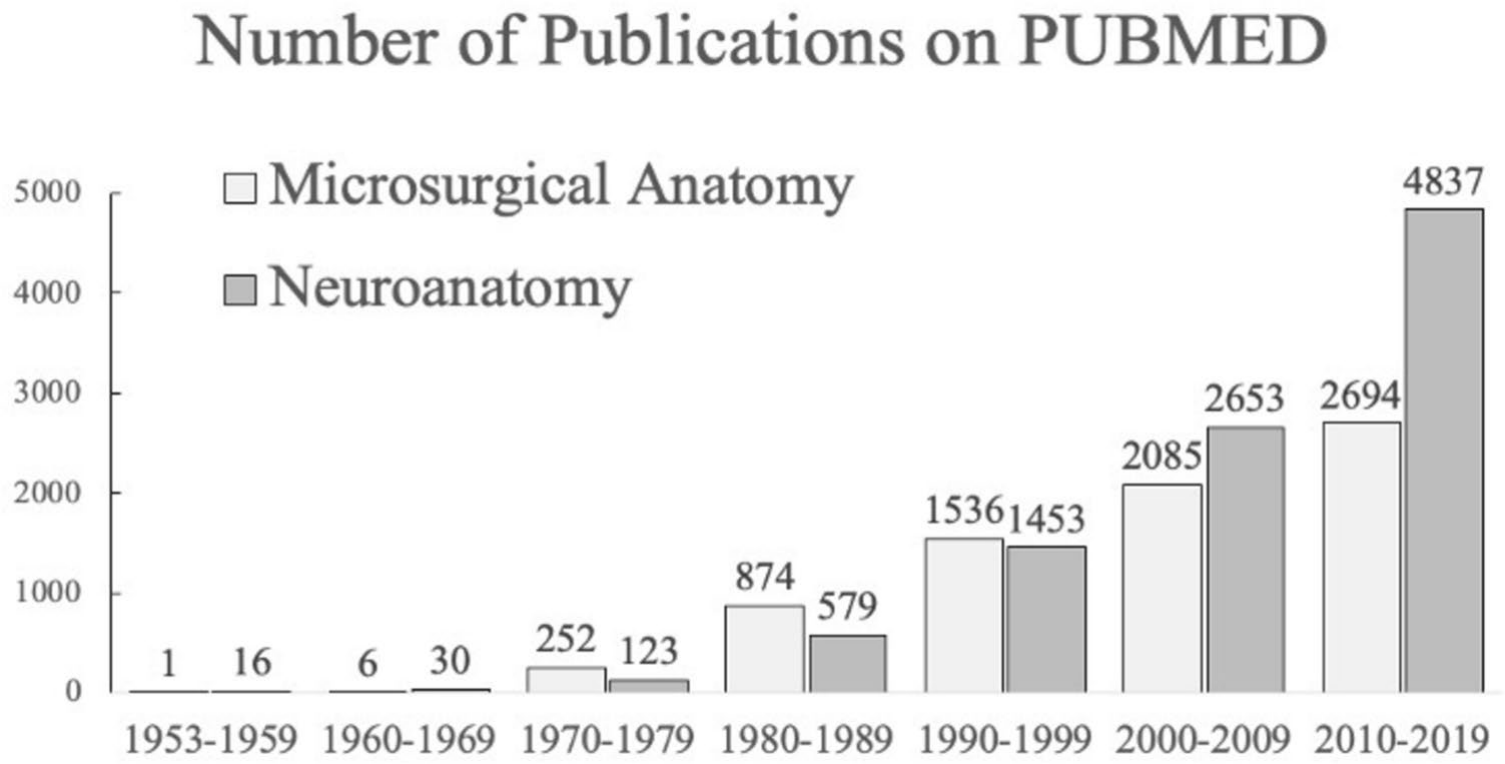
Number of publications on microsurgical anatomy and neuroanatomy

Neurosurgeons have placed great emphasis on microneurosurgical anatomical studies to deepen their understanding of skull base microanatomy. While research on general neuroanatomy has steadily increased, publications specifically focused on microneurosurgical anatomy have grown exponentially (Fig. 1). Pioneering contributions in this field were made by leading anatomists such as Albert Rhoton Jr. [96], M. Gazi Yaşargil [85], and Johannes Lang [80]. These microneuroanatomical investigations led to significant breakthroughs in neurosurgery, particularly in the surgical management of the cavernous sinus.

For many years, the cavernous sinus was regarded as “no man’s land” due to the extremely high risk of complications associated with surgery in this region. The first successful surgical intervention within the cavernous sinus was performed by Jefferson Browder in 1937 [22], but his technique was not widely adopted. A major turning point came with Dwight Parkinson, who conducted over 200 cadaveric dissections of the cavernous sinus and subsequently devised the first direct surgical approach for carotid-cavernous fistulas [12]. His work sparked widespread interest in the surgical treatment of cavernous sinus lesions and underscored the necessity of a comprehensive anatomical understanding before attempting direct interventions. Subsequent anatomical studies by neurosurgeons, including Dwight Parkinson, Jean N. Taptas, Vinco Dolenc, Felix Umansky and Hilel Nathan generated worldwide enthusiasm for a more detailed exploration of the cavernous sinus [32, 115, 141, 144]. This growing body of research culminated in a major landmark when Vinko Dolenc introduced a novel approach to cavernous sinus surgery. By modifying the *pterional approach* [32, 56], he established an extradural corridor to the lateral wall of the cavernous sinus, accessing the region by dissecting between the two dural layers of the lateral wall of the cavernous sinus (after drilling of the lesser wing, opening of the superior orbital fissure, division of the meningo-orbital band, extradural anterior clinoidectomy, drilling of the optic strut and unroofing of the optic canal).This innovative technique, later known as “*Dolenc’s approach*,” allowed for safer and more effective treatment of both neoplastic and vascular lesions within the cavernous sinus. His work reached a broader audience in 1989 when he published a comprehensive book detailing the anatomy and surgical techniques of this complex region, cementing his role as a pioneer in cavernous sinus surgery [148]. Deeper study of the cavernous sinus microsurgical neuroanatomy led Harry van Loeveren and John Tew [36] to define the location of distinct pathologies in the cavernous sinus and relate them to the meningeal architecture [73] and the course of the carotid artery [21] and Shigeaki Kobayashi to elaborate on the microanatomical and histological relations of the carotid artery with the cavernous sinus [76].

#### Novel SBNS approaches were developed

Surgical anatomy became a research field and deeper understanding of microsurgical neuroanatomy played a pivotal role in refining surgical techniques for complex skull base lesions. Neurosurgeons dedicated significant effort to studying the microanatomy of the cavernous sinus, the clivus, and the petroclival region, leading to the introduction of novel surgical strategies. During this period, new skull base surgical approaches emerged, classified based on their anatomical access corridors. These include four main corridors: Anterolateral, lateral, posterolateral approaches and endoscopic anterior midline skull base.

##### The evolution of anterolateral SBNS approaches

**Fig. 2:**
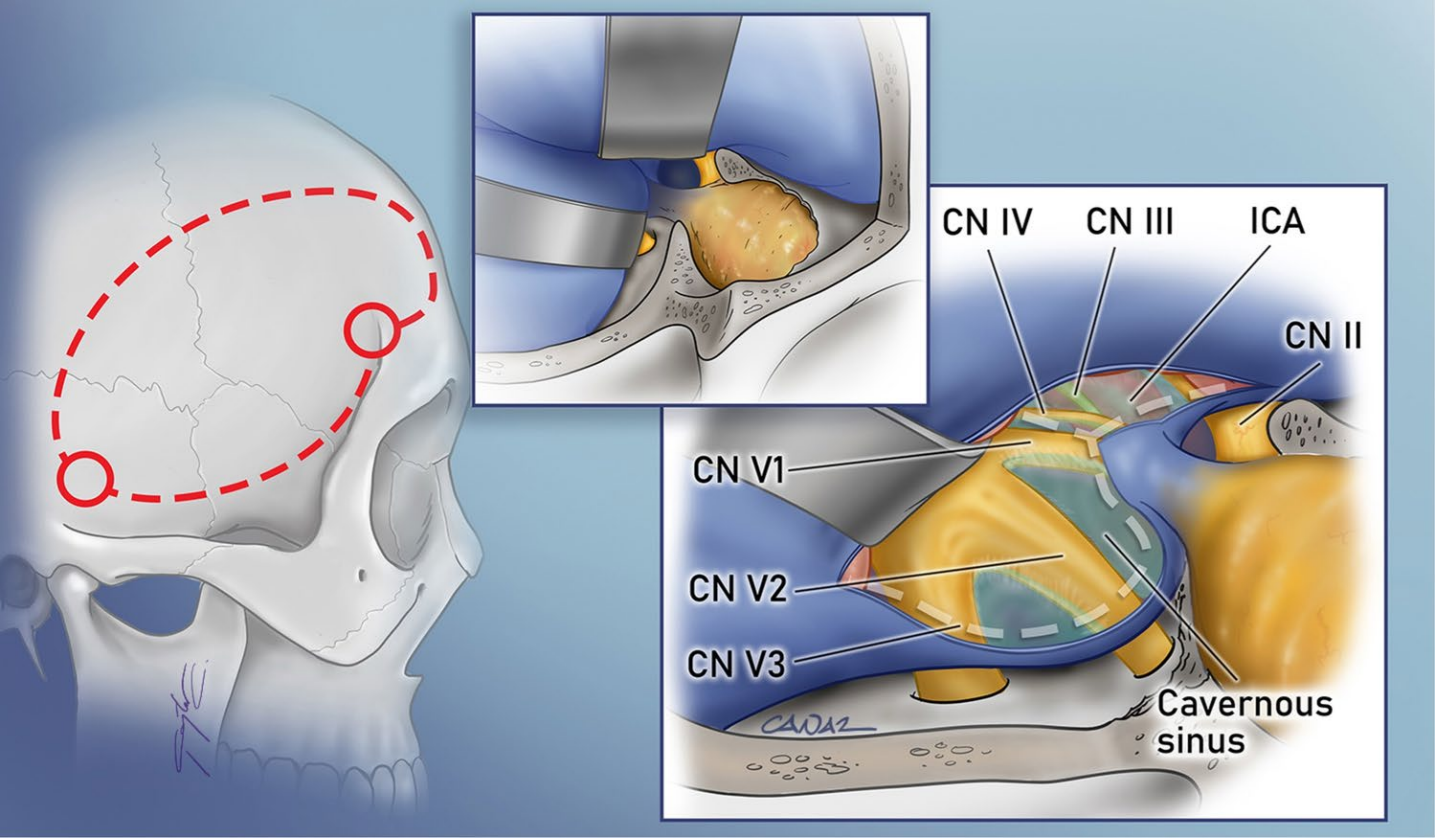
A typical example of the anterolateral approach to the skull base is the Dolenc’s approach. The approach utilizes a pterional craniotomy (marked in red dashed lines in this illustration of a right sided craniotomy). Division of the meningo-orbital band, extradural anterior clinoidectomy, drilling of the optic strut and unroofing of the optic canal is then performed. This is followed by the exposure and splitting of two dural layers at the superior orbital fissure (upper-left inset), allowing for an interdural access to the lateral wall of the cavernous sinus (lower-right inset. The durotomy is marked with a grey dashed lines)

**Fig. 3:**
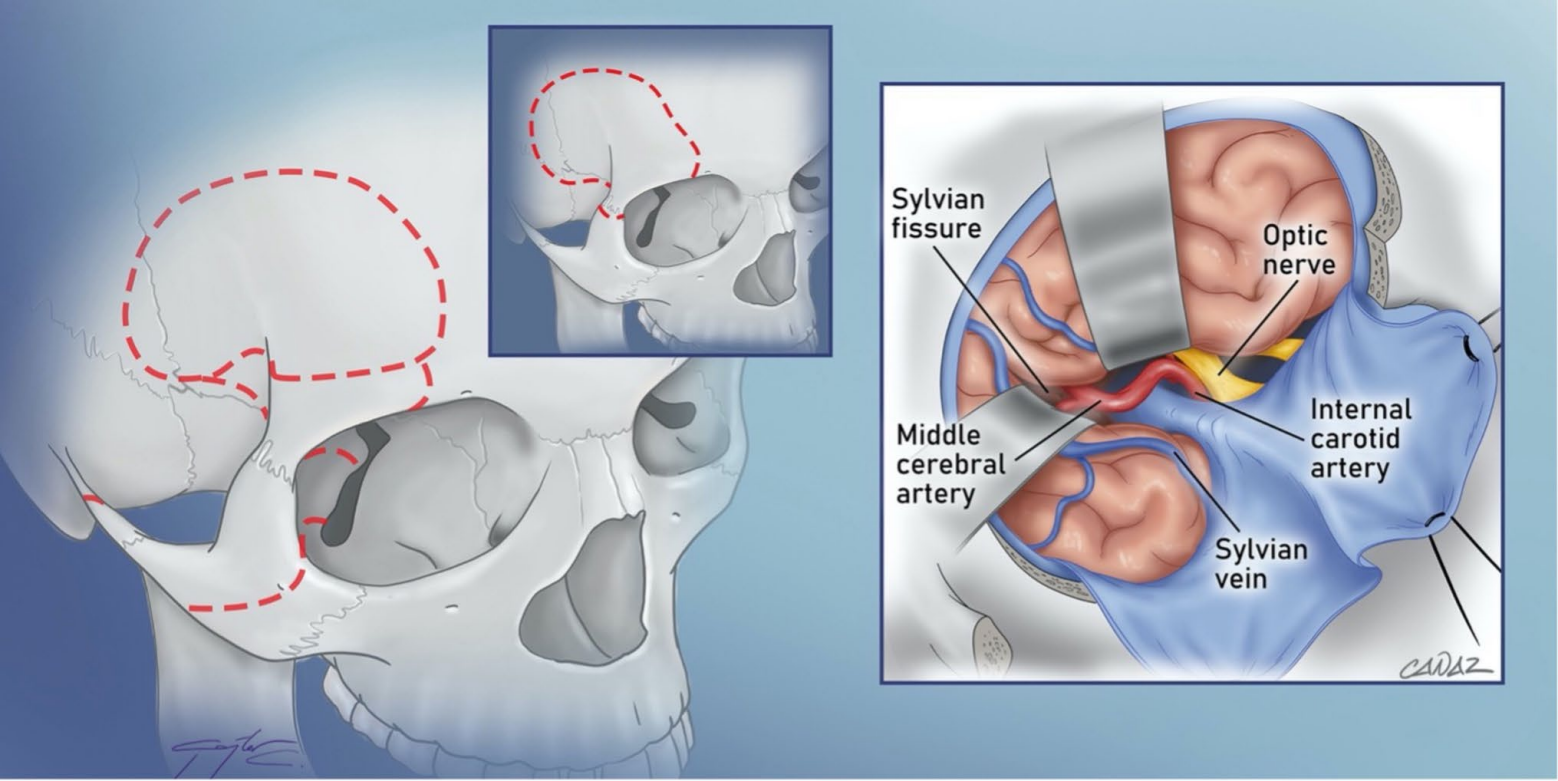
A Cranio-orbitozygomatic (COZ) approach is a modification of the frontotemporal approach and utilizes varying amounts of zygomatic arch and supraorbital bar resections (dashed red lines marking one-piece and multiple piece osteotomies). The approach provides a more cranial view to the brainstem

Anterolateral approaches, such as the *cranio-orbitozygomatic (COZ) approach*, have significantly enhanced exposure to lesions in the anterior and middle cranial fossae, as well as the cavernous sinus. The “*Dolenc approach*”, considered the prototype of these anterolateral approaches, is an extension of the pterional craniotomy. Developed by Vinko Dolenc, it involves a frontotemporal craniotomy, followed by the exposure and splitting of two dural layers at the superior orbital fissure, allowing for interdural access to the lateral wall of the cavernous sinus (CS) [32] (Fig. 2). Several similar interdural approaches have been developed for accessing the lateral wall of the cavernous sinus. Akira Hakuba devised an approach through the dural covering of the maxillary nerve [51], while Atul Goel utilized the mandibular division of the trigeminal nerve [46]. However, limitations in achieving an adequate upward surgical viewing angle over the dorsum sella led to the development of the COZ variation of the anterolateral approach. By incorporating orbital and zygomatic osteotomies, this modification provides a lower basal viewing angle, facilitating superior exposure to high-lying interpeduncular, prepontine, and parasellar pathologies (Fig. 3). A key motivation behind the evolution of the COZ *approach* was the treatment of high-lying basilar tip aneurysms. Until the 1990 s, surgical clipping was the only available treatment for these aneurysms, leading to the emergence of two opposing surgical philosophies. M. Gazi Yaşargil advocated for the *pterional approach* [149]. Charles Drake, on the contrary, favored the *subtemporal approach* [34]. Both methods were effective but had notable limitations: the *pterional approach* was restricted in cases of high-lying basilar tip aneurysms due to its superoinferior viewing angle, whereas the *subtemporal approach* was less suitable for low-lying basilar tip and basilar trunk aneurysms. The *subtemporal approach* also required considerable temporal retraction and the vein of Labbe and the trochlear nerve on the trajectory increased the risk of the approach. These challenges spurred the development of both the COZ and *anterior petrosal approaches*. The COZ approach built upon previous experience with supraorbital rim osteotomies pioneered by Louis Linn McArthur [97], Charles H. Frazier [39], and John A. Jane Jr. [63], A one-piece orbito-zygomatic craniotomy was initially defined for suprasellar lesions and anterior communicating artery aneurysms, with Philippe Pellerin later introducing a separate fronto-orbito-malar osteotomy [117]. Subsequently, Ossama Al-Mefty [2] refined the COZ approach by integrating a fronto-orbito-malar osteotomy with the standard *pterional approach.* To reduce complications and improve cosmetic outcomes, a two-piece technique for the frontotemporal and orbitozygomatic bone flaps was introduced by Marc Sindou and Joseph Zabramski [8, 98, 151]. Later, William Couldwell developed a modified version, which involved removing the lateral orbital rim and wall to enhance access to the anterior cavernous sinus, parasellar region, and middle fossa lesions [10]. This modification aimed to reduce surgical time, minimize brain retraction, and improve postoperative cosmetic results.

##### The evolution of lateral SBNS approaches

**Fig. 4:**
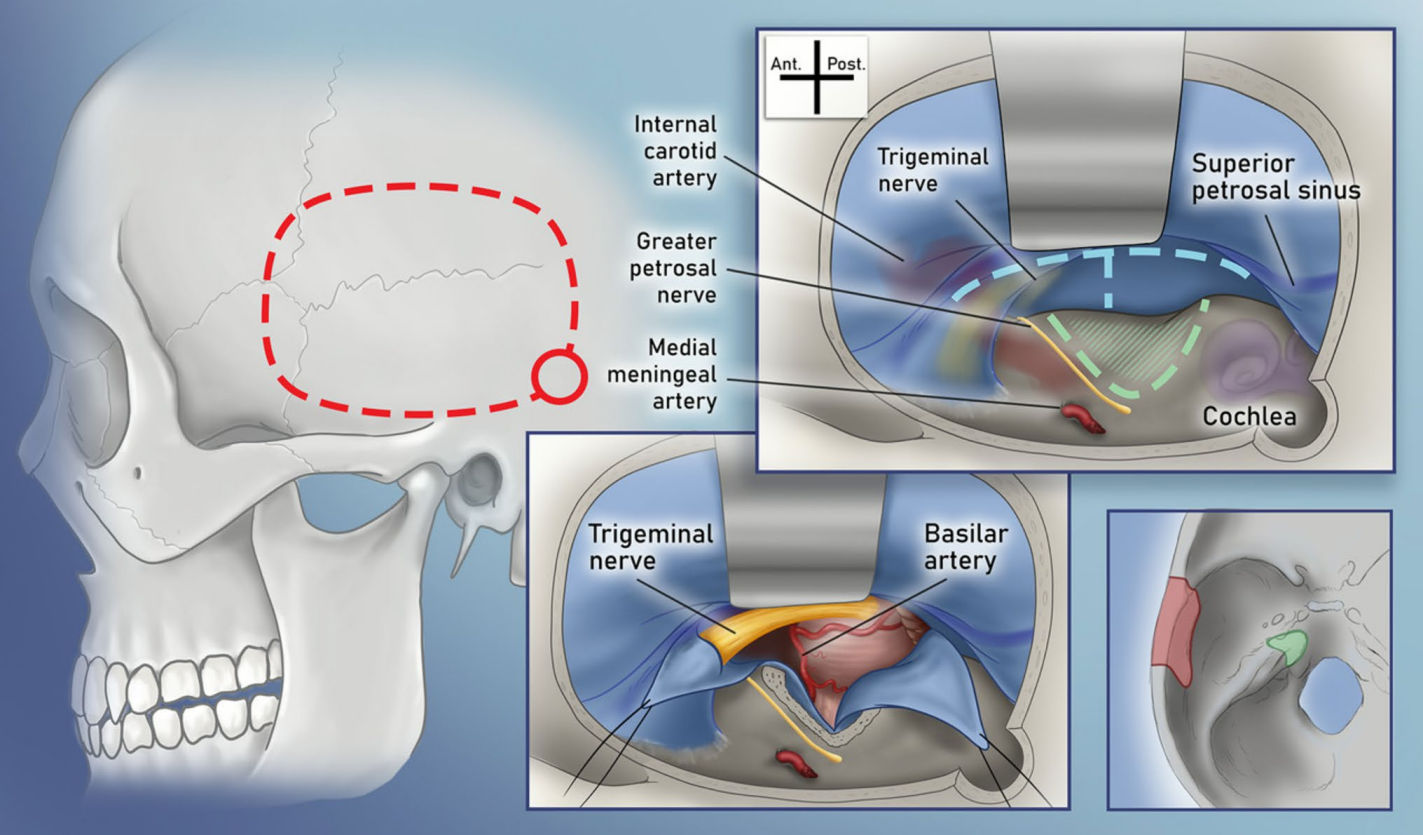
An anterior petrosal craniotomy is used to provide an anterior view to the brainstem. The approach starts with a regular temporal craniotomy (dashed red lines). Drilling of the Kawase’s triangle (green dashed lines between GSPN, arcuate eminence and petrous ridge) between the GSPN and inner ear structures provides removal of a bone portion that is devoid of vital neurovascular structures (marked with green in the lower right inset)

Lateral approaches, including the anterior petrosal and extended middle fossa approaches, have provided safer and more effective access to the petroclival region. Among these, the anterior petrosal approach is the primary lateral technique used in skull base neurosurgery (SBNS). The corridor from the middle fossa to the cerebellopontine angle was first utilized by Robert Henry Parry of Glasgow to divide the auditory nerve for treating tinnitus in 1904 [116]. Later, middle fossa approaches to the internal acoustic canal were developed by William House and Ugo Fisch [61]. In 1973, Andrew W. Morrison and Tom T. King introduced a combined *translabyrinthine* and *transtentorial approach* for large vestibular schwannomas. The approach involved drilling the labyrinthine portion and cutting the tentorium [101]. This technique was further modified in 1975 by otolaryngologists Zbigniew Bochenek and Andrzej Kukwa [19], who extended the approach toward the sigmoid sinus, eliminating the need for the translabyrinthine component. In a further evolution of these methods, Ryuzo Shiobora [69] proposed the “extended middle cranial fossa” approach. Unlike previous techniques, this approach involved both drilling the temporal bone and making an incision in the cerebellar tentorium. However, middle fossa approaches did not gain widespread popularity due to limitations in surgical visualization, particularly when treating larger vestibular schwannomas. Although these techniques had been described earlier, interest in middle fossa approaches was reignited in 1985 by Takeshi Kawase, who had previously collaborated with Shiobora. Kawase refined the subtemporal middle fossa approach to provide access to basilar trunk and tip aneurysms while preserving hearing by avoiding drilling through middle ear structures (Fig. 4) [71]. This approach involved limiting bone drilling to the medial petrous apex, a region now globally recognized as Kawase’s Triangle (Fig. 4). Over time, the technique was further adapted for the surgical management of petroclival meningiomas [47, 72, 129, 132].

Evolution of posterolateral SBNS approaches

##### Evolution of posterolateral SBNS approaches

**Fig. 5:**
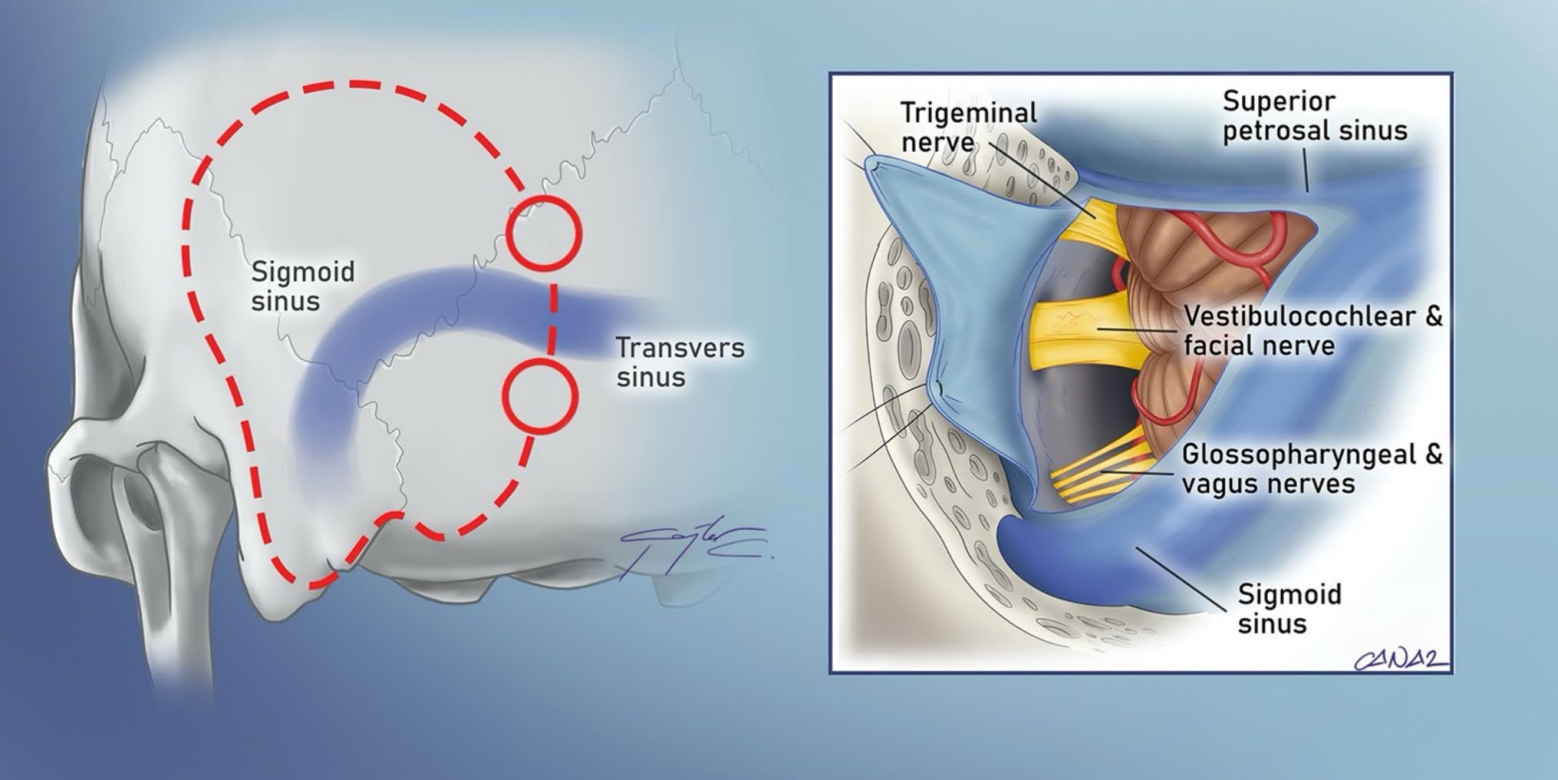
The presigmoid approach is used to gain an anterolateral view to the brainstem. The target are intradural or extradural lesions extending both above and below the tentorium along the petrous ridge, clivus, or both. The approach uses a combination of retrosigmoid and temporal craniotomies on both sides of the transverse-sigmoid venous junction. The petrosal vein is ligated and cut, which allows for posterior mobilization of the venous structures. Temporal bone drilling is done until semicircular canals are skeletonized to protect hearing

**Fig. 6:**
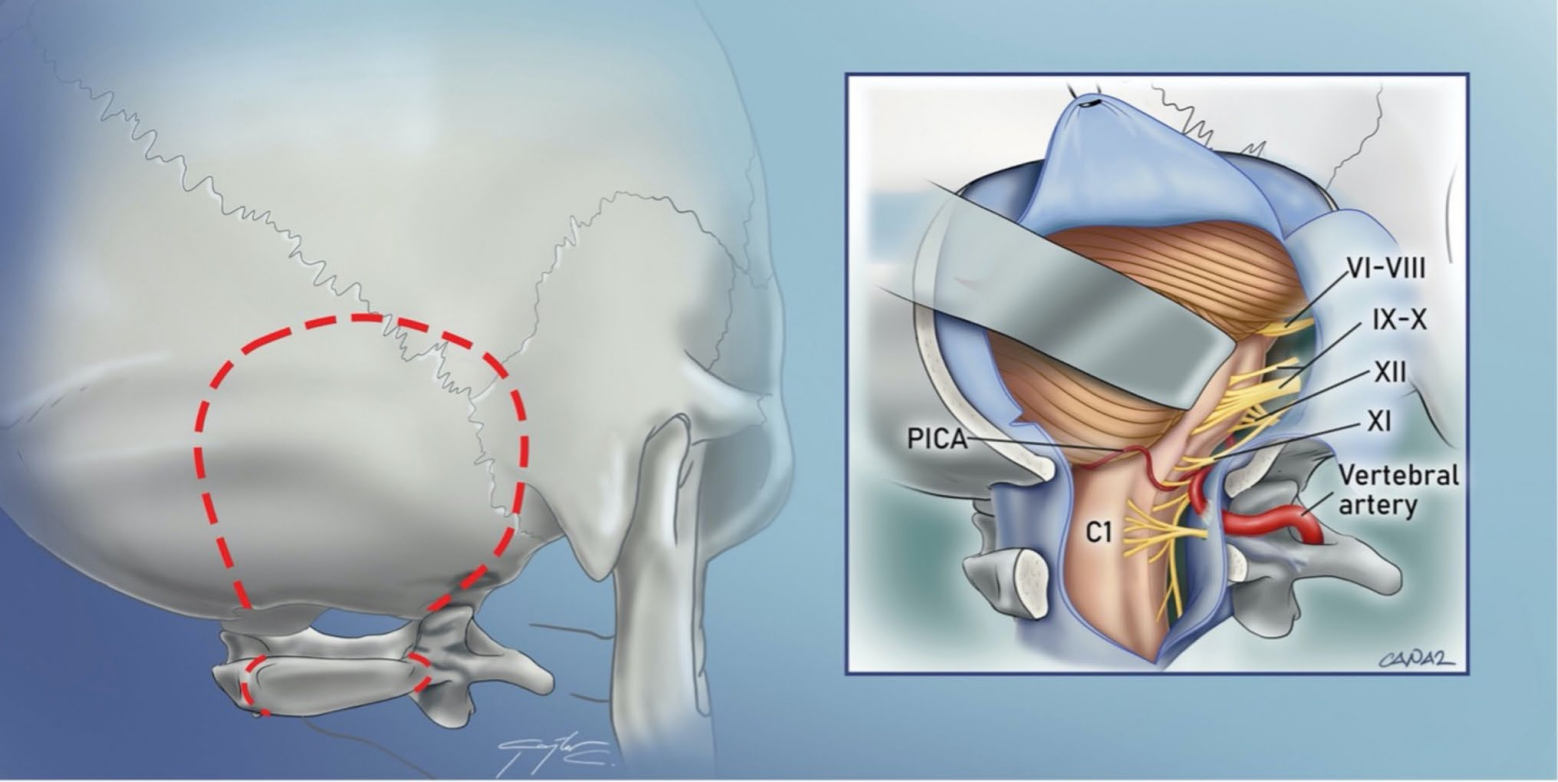
The far lateral approach is a modification of the retrosigmoid craniotomy which is particularly used for lateral approaches to foramen magnum meningiomas and petroclival meningiomas.. It is used to gain an anterior view to the lower brainstem and to provide proximal control of the vertebral artery. The approach utilizes removal of the rim of foramen magnum with or without condylar resection(partial or total), resection of the arch of C1(as marked with red dashed lines) and mobilization of the extradural vertebral artery(inset). Exposure and mobilization of the vertebral artery from the suboccipital triangle are crucial. This is a useful posterior fossa approach

Posterolateral approaches, including the *posterior petrosal/presigmoid* and *far lateral approaches*, have significantly improved surgical visualization of the ventral brainstem and foramen magnum. The *posterior petrosal/presigmoid approach* provides access to the rostral ventral brainstem, while the far lateral approach is primarily used for the caudal ventral brainstem. The various *posterior petrosal/presigmoid approaches* differ mainly in the extent of petrous bone resection. Initially developed by William House as the *translabyrinthine approach*, it was originally designed to expose the facial nerve within the petrous bone for vestibular schwannoma resection [61]. In neurosurgery, these approaches were later adapted for accessing the ventral brainstem, particularly for treating vertebrobasilar aneurysms and clival/petroclival tumors. The resection of the petrous bone, skeletonization of the transverse-sigmoid junction, and ligation or division of the petrosal sinuses allow for posterior mobilization of the venous complex. This creates a posterolateral view of the brainstem and petroclival region, leading to the development of the *presigmoid approach* (Fig. 5). The first descriptions of *transpetrosal approaches* in neurosurgical literature date back to Akira Hakuba in 1977, who introduced them as “transpetrosal surgery for clival meningiomas.” Later, this approach was adapted for craniopharyngioma surgery as the *transpetrosal-transtentorial approach* [52]. However, the widespread adoption of the *posterior petrosal approach* is credited to Ossama Al-Mefty, who in 1988 introduced two key modifications [3]: The first was the skeletonization of the semicircular canals to preserve hearing. The second modification was the posterior mobilization of the sigmoid sinus to expand the surgical viewing angle. These refinements made the posterior petrosal approach highly effective, allowing exposure approximately 3 cm closer to the tumor and at least 2 cm more anteriorly. Over time, several neurosurgeons have contributed further modifications, including Madjid Samii [122], Laligham Sekhar [65] [131], and Takanori Fukushima [41]. However, challenges such as prolonged surgical duration, ipsilateral hearing loss, and the risk of Labbe venous infarctions remain significant limitations. Therefore modifications have been devised to limit the extent of the approach, shorten it and decrease the risk [142]; among other modifications a more medial dural opening reduces risks of damage to the tentorial draining veins. The *far lateral or the extreme lateral approach* provides a posterolateral surgical corridor to access the ventral foramen magnum. Initially, neurosurgeons attempted *transoral, transbasal, and transmaxillary approaches* for lesions in the inferior third of the clivus [103]. However, due to poor surgical outcomes and a high risk of infection, posterior fossa approaches became the preferred alternative. The term "extreme lateral (removal of bone)" was introduced by Roberto Heros for targeting vertebrobasilar aneurysms [58]. This technique involves removal of the inferolateral rim of the foramen magnum to the level of the condylar fossa to gain a lateral view to the premedullary cistern. The approach requires mobilization of the extradural vertebral artery to gain proximal control. The third defining characteristic is the removal of the arch of C1 on one side to gain caudocranial view of the vertebrobasillary junction with no brainstem retraction (Fig. 6). By allowing lateral mobilization, this approach facilitates access to anterior lesions, improving reach to anteriorly extending tumors. The approach had been described initially sketched by Wolfgang Seeger in his 1980 book [17, 127]. Various modifications of condylar resections (one-third, one-half, and two-thirds condylectomy) have been introduced to expand the surgical field and enhance visibility. Refinements of the approach were described by Helmut Bertalanffy and Wolfgang Seeger [17], Axel Perneczky [108], Robert Spetzler [29], Bernard George [43], Akira Hakuba [55] and Takanori Fukushima [40]. Later, Felix Umansky described a modification allowing for lateral mobilization of the vertebral artery, which was named the “*lazy far lateral*” approach due to the shape of the incision [102]. More extensive lateral variations involving greater occipital condyle and lateral mass drilling have been described, but these often come at the cost of cranio-cervical instability [44, 133]. To optimize patient outcomes, preoperative evaluation of the foramen magnum shape is essential for determining the necessary extent of condylar resection. Recent studies have suggested that extensive condylar resection may not always be necessary, as space-occupying lesions naturally displace the brainstem [15].

##### Combined Approaches: Overcoming Exposure Limitations

As SBNS evolved, limitations in individual approaches were often addressed by combining multiple techniques. In 1970, Andrew W. Morrison and Tom T. King [74] combined the *translabyrinthine* and *transtentorial approaches* to widen the exposure to the posterior fossa and upper brainstem, complementing the middle fossa approach. A critical feature of these combined approaches is the tentorial division, which facilitates superior access. Zbigniew Bochenek and Andrzej Kukwa [19] described a similar combination technique. By 1978, Akira Hakuba [53] had adapted these combined approaches for clivus meningiomas, while later refinements were made by pioneers such as Takanori Fukushima [78], Laligham Sekhar [132] and Ossama Al-Mefty [25]. A comprehensive list of skull base approaches, their pioneers, and initial uses is provided in Table 1.

**Table 1:** Skull base approaches with their initial use and pioneers

Approach direction	Approach class	Initial use and Pioneers
Anterior Approaches	**Transsphenoidal & extended transsphenoidal app**	**Transsphenoidal** Schloffer 1906 [153],Cushing 1909,Kocher 1909, Hirsch 1910, Halstead 1910, Chiari 1912, Preysing 1913, Dott 1923, Guiot 1957, Hardy 1967 [84] **Extended TS** Hirschmann 1901, Dandy 1922, Guiot 1962,Laws,1982, Weiss 1987,Kassam-Synderman 2001 [154]
	**Other transfacial app**	Trans basal: Derome 1985 [155] Transethmoidal: Moure, 1922 [156] Trans maxillary: Cheever 1867 [157] Trans mandibular: Krespi & Sisson 1984 [158] Mandibular Splitting: Drommer,1986 [159] Transoral: Crockard,1988 [26]
Anterolateral Approaches	**Cranio-orbito-zygomatic**	McArthur 1912 [97], Frazier 1913 [39], Jane 1982 [63], Pellerin 1984 [117], Al-Mefty (1987) [2]
	**Interdural app. to cavernous sinus**	Dolenc 1983 [32], Parkinson 1965 [115], Kawase (1985), Umansky& Nathan 1982 [144],Taptas 1982 [141], Hakuba 1989 [51], Goel 1995 [46],
Lateral Approaches	**Anterior petrosal approach**	**Anterior petrosal approach** Kawase-1985 [71], House-1961 [61]
		**Infratemporal fossa approaches** Fisch & Pillsbury-1979 []^38^
	**Posterior petrosal (presigmoid) approaches**	**Translabyrinthine** House (1964) [60]
		**Retro Labyrinthine/transpetrosal/presigmoid** Hakuba & Nishimura 1985 [160] Kishi, Nakamura (1977) Al-mefty (1988) [3] Samii 1988 [122] Sekhar 1991 [65] Fukushima [41]
		**Total Petrosectomy (transcochlear)** Lempert (1937) [161], House& Hitselberger (1966) [162]
Far Lateral Approaches		**Far Lateral** Koos 1985 [163], Heros 1986 [58],Pernezcky 1986 [108], Bertalanffy&Seeger 1991 [17], Spetzler 1998 [29], George& Lot 1995 [43], Hakuba1993 [55], Fukushima 1996 [40]
Major Combinations		King, Morrison 1970 [74], Bochenek, Kukwa 1975 [19] Sekhar,Schessel 1999 [132] Al-Mefty, Cho 2002 [25]

##### Evolution of anterior approaches and development of skull base endoscopy

Endoscopic endonasal approaches have gained significant traction, particularly for parasellar and clival tumors. The *transsphenoidal approach* to the cranial base originally began with pituitary adenoma surgery. Until the 1990 s, microscopic transsphenoidal surgery was the standard technique. In the early 1990 s, the rise of Functional Endoscopic Sinus Surgery (FESS) revolutionized the field. ENT surgeons demonstrated that endoscopic techniques provided superior visualization and excellent surgical outcomes. This success led to an increasing adoption of endoscopy in skull base surgery. Initially, the endoscope was used as an adjunct to the microscope, a technique known as "endoscope-assisted transsphenoidal surgery." In the 1990’s otolaryngologist, Roger Jankowski performed the first pure endoscopic approach to the sella [64].This was followed by the rapid development of the pure endoscopic pituitary and skull base surgery by neurosurgical pioneers including Hae-Dong Jho [66] and Paulo Cappabianca & Luigi Cavallo [23]. In the early years of pure endoscopic skull base surgery, cerebrospinal fluid leaks were a very significant problem. A cornerstone technique for limiting CSF leaks was the introduction of the nasoseptal flap by Gustavo Haddad [50] and its popularization by Amin B Kassam and Paul Gardner [70]. These reconstruction techniques dramatically reduced the incidence of postoperative CSF fistula, which previously exceeded 10% and remained the most common complication in endoscopic skull base surgery. The introduction of the “gasket-seal” closure further decreased CSF leak rates [42, 82]. Subsequent refinements led to the establishment of a multilayered closure algorithm, combining inlay dural substitutes, fascia or fat grafts, and vascularized flaps, thereby forming an evidence-based reconstructive ladder. Currently, various multilayered repair techniques including the bilayer-button [86], in-situ bone flap [68], and 3 F technique [24] have been proposed and have further reduced the incidence of postoperative CSF leakage. After better controlling for the risk of CSF leak pure endoscopic techniques had become the standard for transsphenoidal surgery in 2020’s. The enhanced illumination, visualization, and maneuverability offered by the endoscope expanded surgical access beyond pituitary adenomas, allowing for procedures that were previously impossible with the microscope. These advancements made possible a transsphenoidal access to the anterior cranial fossa [67], medial decompression of the optic canal [16], medial exploration of the cavernous sinus [37], exploration of the third ventricle through parasellar corridors [4], complete endoscopic removal of the clivus [4]. Such advantages facilitated the use of endoscopic skull base surgery and made it into a cornerstone of modern skull base procedures.

#### Characteristics of distinct skull base pathologies were better understood

Advances in microsurgical neuroanatomy and the development of novel surgical approaches have significantly enhanced our understanding of skull base pathologies. Two prime examples are the progress is the evolving knowledge of clinoidal meningiomas and tuberculum sella (TS) meningiomas:

Clinoidal meningiomas, or meningiomas arising from the inner third of the sphenoid wing, around the anterior clinoid process, were first described by Harvey Cushing in 1938 [99]. He categorized them as a subgroup of sphenoid wing meningiomas, referring to them as “those of the deep or clinoidal third” [100]. Later, Clovis Vincent introduced the term “spheno-cavernous meningiomas” [14]. In 1980, Joel Bonnal classified clinoidal meningiomas as Group A among sphenoid wing meningiomas based on tumor extension [20]. A decade later, Al-Mefty refined this classification, distinguishing clinoidal meningiomas from inner-third sphenoid wing meningiomas and organizing them into three subgroups based on the point of origin [5]. Clinoidal meningiomas form a distinct entity separate from medial sphenoid wing meningiomas. Radiologically, they are characterized by an upward growth pattern arising from a small dural attachment on the anterior clinoid process, often accompanied by hyperostosis evident on computed tomography (CT). Unlike medial sphenoid wing meningiomas, clinoidal variants seldom invade the cavernous sinus and typically cause only minimal compression of the optic nerve, with optic nerve sheath involvement being exceptionally rare. In contrast, meningiomas of the medial third of the sphenoid wing typically grow toward the anterior aspect of the medial temporal lobe. For accurate preoperative differentiation, coronal CT imaging is recommended for any suspected meningioma. This classification has been widely adopted worldwide. Based on our research, we propose incorporating tumor size into the existing classification system [114].

Another critical example is tuberculum sellae (TS) meningiomas. Over the years, numerous studies have demonstrated a high incidence of optic canal involvement in these tumors [111]. TS meningiomas frequently extend into the optic canal, either unilaterally or bilaterally, often presenting with asymmetric visual deficits, which serve as a key diagnostic indicator. This understanding has led to the routine incorporation of optic canal decompression with intradural or extradural drilling of the anterior clinoid process. These modifications to the routine pterional craniotomy have significantly improved both immediate and long-term surgical outcomes [88, 94, 121].

#### The outcome of SBS improved significantly over time

It did not take the neurosurgical community very long to realize that SBNS procedures had superior surgical outcomes when compared to standard microneurosurgery. Obvious examples are numerous but only two pathologies will be demonstrated here: Ventral foramen magnum meningiomas and trigeminal schwannomas.

Compared to other meningiomas, ventral foramen magnum meningiomas were considered to have the worst surgical outcomes. After the development of SBNS, the gross total resection rates for these tumors have increased nearly to 100% and surgical mortality rates have decreased nearly to 0%. In one of our studies, we showed the success of *far lateral approach* in ventrally located foramen magnum meningiomas [11, 18, 26, 45, 48, 90, 110, 124, 133, 150].

Another striking example is trigeminal schwannoma surgery. Anatomical studies on cavernous sinus have led to the reclassification of trigeminal schwannomas as interdural tumors of the lateral cavernous sinus wall and opened the way for development of various interdural microsurgical and endoscopic SBNS approaches. With these advances, GTR rates have increased nearly to 100% [6, 91, 113, 125]. Beyond achieving tumor control, modern skull base surgery increasingly emphasizes postoperative quality of life (QOL) as a key outcome measure. Studies show that most patients experience good long-term QOL, with gradual improvement during the first postoperative year. Factors negatively affecting QOL include malignancy, older age, comorbidities, adjuvant radiotherapy, and extensive surgery. Multidisciplinary care, tailored pain management, and psychological or group-support interventions have been shown to further enhance recovery and long-term well-being. As survival outcomes converge across techniques, QOL has become a central benchmark for assessing the true success of SBNS [75, 128, 138].

### The fourth stage: maturation of SBNS

**Fig. 7:**
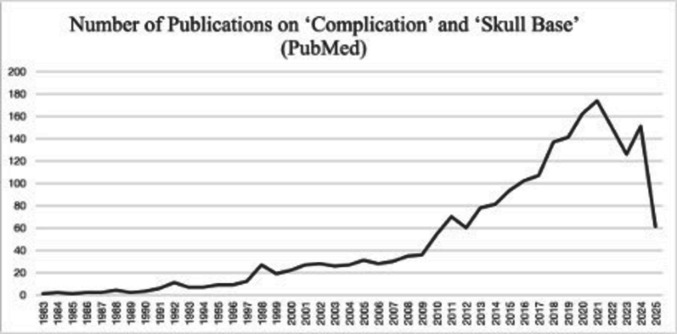
A Pubmed search with the keywords (“complication” AND “skull base”) clearly indicates to a better understanding of complications and a focus on complication avoidance

The maturation of SBNS was marked by a critical re-evaluation of outcomes and an emphasis on optimizing surgical strategies. During the early days of SBNS, each novel approach was met with enthusiasm and initial publications usually reported very high success rates. This surely fueled the popularization. However, later reports, with ever growing cohorts, failed to reproduce the success of initial reports. Dramatic examples are cavernous sinus and petroclival meningiomas where initial studies reported very high resection rates but later studies in far larger cohorts and improved radiological standards resulted in a progressive drop in reported total resection rates in cavernous sinus meningiomas [1, 7, 59, 130, 134], or petroclival meningiomas [3, 13, 54, 106]. Reported gross total resection rates in cavernous sinus meningiomas. Also the cranial nerve morbidity and other complications were more and more recognized and admitted. Long-term follow-up studies revealed that, while aggressive resection strategies initially appeared successful, they often led to significant morbidity, particularly in cases involving cranial nerve involvement and did not necessarily prevent recurrences [30, 83, 105] Importantly, the potential suffering of very extensive surgery could be overshadowed by the surgeon’s enthusiasm [81, 95]. As a result, a paradigm shift occurred, favoring function-preserving approaches or limited approaches when possible [13, 128] (Fig. 7).

#### The role of gamma knife radiosurgery

The introduction of Gamma Knife radiosurgery as a neurosurgical tool played a transformative role in SBNS. Initially developed by Lars Leksell in the late 1960 s, this non-invasive treatment modality became increasingly utilized in the 2000 s as an adjunct to surgery. Skull base tumors such as vestibular schwannomas, cavernous sinus meningiomas, and clival chordomas were effectively managed with radiosurgery, either as a primary treatment or following subtotal resection. This approach allowed for tumor control with minimal collateral damage, reducing the need for high-risk surgical interventions [77].Tiit Mathiesen proposed the “planned” subtotal tumor removal of a meningiomas followed by radiosurgery and named it the "Simpson 4-Gamma" concept [95]. This approach achieved long term recurrence rates comparable to those of a Simpson grade 1 resection but with significantly lower morbidity [57, 112, 140]. This strategy proved particularly beneficial for benign tumors of the skull base, reinforcing the shift toward safer, multimodal treatment approaches.

#### The rise of endoscopic SBNS

Endoscopic skull base surgery saw significant advancements in the 2000 s, particularly in the management of midline skull base tumors. Initially pioneered by Gerald Guiot in the 1960 s but abandoned due to technological limitations, endoscopic techniques gained widespread adoption with the advent of high-definition optics, improved instrumentation, and navigation-assisted surgery.

Classical approaches to address midline skull base tumors such as the *transbasal approach* of Patrick J. Derome, the *transmaxillary approach*, mandibulary splitting, *transoral approach* or resection of the palate became less and less commonly used. Endoscopic endonasal approaches became the preferred method for addressing pituitary adenomas, craniopharyngiomas, and clival chordomas. Although the endoscopic endonasal approach (EEA) has gained popularity in recent decades for the management of midline skull base lesions, its superiority over traditional transcranial approaches remains controversial. Comparative studies and meta-analyses have shown no consistent differences in gross total resection or overall complication rates between the two techniques [79, 87, 92, 104, 118, 120, 145]. Each approach carries distinct advantages and limitations, and surgical expertise remains a critical determinant of outcome. The introduction of EEA has markedly facilitated the resection of midline skull base lesions by providing a direct, ventral corridor with minimal brain retraction, establishing it as a valuable alternative to open microscopic-microsurgical techniques within the armamentarium of skull base surgery.

#### Refinement of surgical technique and treatment strategies

The ongoing evolution of SBNS has led to a continuous refinement of surgical approaches and treatment indications. Traditional extensive skull base approaches have been modified to prioritize safety, efficiency, and functional preservation. To name a few: There was a marked shift from extensive craniotomies to more tailored keyhole approaches (as seen in the COZ and *presigmoid approaches*). Similarly, *“small*” or *"lazy" far lateral approaches* reduced morbidity in foramen magnum surgeries [142]. *Retrosigmoid approaches* for vestibular schwannomas were modified to include drilling of the acoustic meatus to enhance hearing preservation. Endoscopy was combined with standard approaches for early identification of tumor remnants to result in higher gros total resection rates. Finally, Gamma Knife radiosurgery, which was invented as a neurosurgical tool, was commonly added to treatment strategies. The combination of maximal safe resection with adjuvant Gamma Knife treatment to achieve symptom relief and local tumor control for skull base meningiomas (Simpson-4 Gamma) [95].

#### Increased academic efforts, international collaborations, and education

As SBNS matured, international collaboration and knowledge dissemination became central to its continued progress. The establishment of dedicated skull base surgery societies, training programs, and international courses allowed for standardized education and skill development. Key milestones included the founding of the World Federation of Skull Base Societies (WFSBS) and regional skull base societies; popularization of cadaver-based dissection courses to enhance surgical training; the publication of numerous specialized textbooks and journals which focused on skull base techniques and very importantly, increased collaboration between neurosurgeons and other specialists, fostering a multidisciplinary approach to skull base pathology management. Today, SBNS is performed in most large neurosurgical centers around the world. Maturation of the field also has led to standardization of the most basic skull base approaches internationally. This was due to a close collaboration of neurosurgeons interested in SBNS internationally. Since the organization of the first international meeting by Madjid Samii in 1992 international meetings on SBNS became routine. National skull base societies quickly became international and scientific journals emerged. Various local and international societies worldwide are promoting the refinement of SBNS, teaching and scientific publication. The first of these groups was founded by Pierre Rabischong in 1979 under the name of Skull Base Study Group. In 1982 the first Neurological Surgery of the Ear and Skull Base Conference was held in Los Angeles in 1982. The International Skull Base Society was founded in Zurich during a meeting arranged by Ugo Fisch in 1988. In the following years, the North American Skull Base Society was founded, and the first president was Paul Donald [49].In addition, WFNS SBS Committee was established to organize skull base courses in 2001 and the  2002 and then M. Necmettin Pamir between 2009–2013. They performed numerous cadaver courses over the world to educate young neurosurgeons on SBNS approaches. A list of current societies and their respective foundation dates are presented in Table 2*.* These meetings and associations increased international collaboration and enabled publications. Again, thanks to these collaborations, neuroanatomy courses were organized to introduce SBS techniques to the whole world.

**Table 2:** International associations of skull base surgery

Name(Alphabetical)	Founded	Web-site
Asian-Oceanian Skull Base Societies	1989	
Brazilian Skull Base Society		
British Skull Base Society	1992	www.skullbase.co.uk
China Skull Base Surgery Multidisciplinary Collaboration		
Deutsche Gesellschaft für Schaedelbasischirurgie	1993	www.dgsb.de
Egyptian Society of Skull Base Surgery	2008	www.skullbasesurgery-eg.org
European Skull Base Society	1993	www.esbs.eu
Italian Skull Base Society	1991	www.societabasicranio.it
Japanese Society for Skull Base Surgery	1989	
Skull Base Society—Turkey	2019	www.kafatabanidernegi.org
Korean Skull Base Society	1994	www.skullbase.or.kr
Latin American Federation of Neurosurgical Societies – Chapter of Skull Base		
North American Skull Base Society	1989	www.nasbs.org
Skull Base Society of India	1998	www.sbssi.org
World Federation of Skull Base Societies	1988	www.wfns.org
World Federation of Neurological Societies- Skull Base Surgery Committee	2001	www.wfns.org

## Discussion

Over several decades, SBNS has revolutionized the treatment of complex neurosurgical pathologies, significantly improving patient outcomes. Its evolution from rudimentary early attempts to a refined and sophisticated discipline highlights the remarkable advancements in microsurgical neuroanatomy, surgical technology, and multidisciplinary collaboration.

Our narrative reveals a clear picture of organic development where small innovative improvements have opened new ways to address the skull base and it’s pathologies. The development shows a dialectic structure where previously as “inoperable lesions” could be attacked with new methods to achieve better tumor control, but where the new approaches led undesirable side effects, again supporting a retreat to less aggressive goals; in the next stage loss of lesion control fueled renewed attempts to control lesions. In this organic development, a network of pioneers and peers has been indispensable and the SBNS has developed as a collective, collaborative effort. Moreover, the dependance on skull-base microsurgery as a craft has fostered a culture where disciples learned from masters. SBNS requires theoretical as well as practical skill and safe surgery has largely relied on practical training.

The maturation of SBNS has led to the standardization of many approaches, integration of minimally invasive techniques, and adoption of multimodal treatment strategies, ensuring safer and more effective care for patients. SBNS has become an essential part of the core curriculum in neurosurgical training; training in SBNS continues to improve with hands-on courses, development of technological adjuncts and structured programs. Surgical planning can by individualized and integrated with simulation. Despite these achievements, the field continues to evolve, driven by innovations in surgical navigation, robotics, and biological therapies.

In the fourth phase we described the observations of patient related outcomes and described endoscopy and radiosurgery as means to optimize outcomes, albeit possibly at the expense of extent of removal of neoplastic processes. It is probable that future development will again focus on extent of removal and long-term control with continued observation of issues relevant for long-term quality of life.

As we look toward the future, SBNS remains at the forefront of neurosurgical innovation, with ongoing research and technological advancements promising even greater strides in patient care and surgical excellence paired with dissemination to provide once unique services to a large population.

## Conclusion

SBNS did not result as a single paradigmatic scientific revolution but emerged organically within neurosurgery as a means to address challenging skull base pathologies and has evolved to become an integral part of neurosurgery.

## Data Availability

No datasets were generated or analysed during the current study.
